# IN-HOSPITAL INSTRUMENTAL CARE PROVIDED BY FAMILY AND STAFF AND HOSPITALIZATION OUTCOMES OF OLDER ADULTS

**DOI:** 10.1093/geroni/igad104.2168

**Published:** 2023-12-21

**Authors:** Ksenya Shulyaev, Nurit Gur-Yaish, Efrat Shadmi, Anna Zisberg

**Affiliations:** University of Haifa, Haifa, Hefa, Israel; Oranim Academic College of Education, Tivon, HaZafon, Israel; University of Haifa, Haifa, Hefa, Israel; University of Haifa, Haifa, Hefa, Israel

## Abstract

Most of hospitalized older adults receive instrumental care (such as help with dressing, bathing, eating) from hospital staff and family members. Current study explored effect of family and staff involvement in this kind of care on hospitalization outcomes. Study sample included 615 acute hospitalized older adults (Age 77.6± 6.7, females 44.3%). Data about patients’ independence in Basic Activity of Daily Living (BADL), level of depression and anxiety was collected twice: at admission to hospital and at discharge. Sociodemographic (age, sex, family status and number of children) and health (severity of illness, comorbidity, length of stay and cognitive status) characteristics, were collected at admission and from medical records. Involvement of family and staff in instrumental care during hospitalization was reported by patients at discharge. The multivariate regressions, which included all the covariates mentioned above, revealed that instrumental care provided by family and by hospital staff significantly predicted hospitalization outcomes, yet in opposite directions. Specifically, family involvement in instrumental support was associated with lower BADL recovery (β=-.36, p<.001), as well as increased depression levels (β=.13, p=.002) and anxiety (β=.17, p<.001). In contrast, instrumental care provided by staff was associated with better BADL recovery (β=.24, p<.001), as well as decreased depression (β=-.21, p<.001) and anxiety (β=-.11, p=.013). These findings highlight the importance of effectively balancing family and staff involvement in instrumental care to optimize patient recovery, improve patients’ outcomes and ensure that they receive the best possible care during their hospital stay.

